# Evaluation of the efficiency and safety of botulinum toxin A injection on improving facial scars: A protocol for systematic review and meta-analysis

**DOI:** 10.1097/MD.0000000000023034

**Published:** 2021-01-08

**Authors:** Dan Xu, Da-Song Zhang, Xue-Feng Hu, Meng-Yao Hu

**Affiliations:** a Plastic Surgery, Wuhan Third Hospital, Wuhan; b National Engineering Research Center for Biomaterials, Sichuan University, Chengdu, China; c Department of Dermatology, Huangshi Central Hospital, Affiliated Hospital of Hubei Polytechnic University, Edong Healthcare Group; Hubei Key Laboratory of Kidney Disease Pathogenesis and Intervention.

**Keywords:** facial scars, botulinum toxin A, efficacy, safety, meta-analysis

## Abstract

**Background:**

Botulinum toxin A injection is an established method of treatment. Clinical practitioners use it widely in their practice to prevent the occurrence of facial scars. However, the effectiveness and safeness of has not been comprehensively established. The objective of the current systematic review is to evaluate the efficacy and safety of using botulinum toxin A injection to improve facial scars.

**Methods and analysis:**

This systematic review involves browsing a number of electronic databases to search for related articles. The search will include databases in both English (PubMed, EMBASE, Web of Science, Spocus, and Cochrane Central Register of Controlled Trials) and Chinese (WanFang database, China Nation Knowledge Infrastructure, and VIP database), the periods of searching will be from inception till the 15th of September 2020. Completing the search in databases allows to consider randomized controlled studies that compares botulinum toxin A interventions to any comparison interventions in those who have facial scars. The review will be inclusive of papers in both languages, English and Chinese. The independent screening of studies for eligibility is conducted by 2 independent authors. Discussion was used to resolve discrepancies between the authors. The Cochrane Risk of Bias Tool V.2.0 is adopted for evaluating the methodological quality of each study. Data extraction was performed by 2 independent authors. For dichotomous outcomes, the were expressed as relative risk (RR) with 95% confidence intervals (CI). For continuous outcomes the results were expressed as the mean difference (MD) or standardized mean difference (SMD) with 95% CI. The statistical analysis of the present study is carried out in RevMan 5.3 software.

**Results:**

This study will output a comprehensive synthesis of existing evidence in relation to botulinum toxin A. Moreover, the results will also provide an interpretation of the effectiveness and safety of botulinum toxin A.

**Conclusion:**

The present review contributes to the existing body of knowledge by adding more evidence to evaluate if botulinum toxin A is effective and safe to be used as an intervention for improving facial scars.

**OSF registration number:**

DOI 10.17605/OSF.IO/94TXP (https://osf.io/94TXP/).

## 1 Introduction

Aesthetic disfigurement is primarily caused by facial scars. Patients who have facial scars tend to be distressed, since it affects their psychology. In addition to the physical pain, the mental strain caused by the distress reduces the self-esteem and confidence of patients, they have an insecure image of their body, patients could also perform poorly at work, facial scars are a condition that diminishes the overall life standard of patients.^[1,2]^ Once the skin is damaged to the reticular dermis, scarring occurs as a natural process to heal the damage inflicted on the skin. There are many social stereotypes associated with scars, certain scars are accepted in society, even admired. However, when someone has a scar on their face, it is viewed as a disfigurement, and labelled as ugly. There are many causes for scars, such as, wounds, burns, trauma, congenital, infections, acne, and surgical excision.^[3,4]^ Comprehending the root cause of a specific wound or scar is vital for the appropriate management and optimizing of cosmetic and functional outcomes. Over the years, numerous non-operative methodologies have progressed together with the unprecedented advancement of technology and aesthetic and dermatologic surgery to camouflage facial scars that are immediately noticeable.

Botulinum toxin is a neurotoxin produced by Clostridium botulinum. It can interfere with the release of acetylcholine from the presynaptic membrane of peripheral motor nerve terminals, causing muscle relaxation and inhibiting excessive sweating. Injecting botulinum toxin is widely adopted to rectify disfigurements or mitigate the development of rhytids, mostly in the upper third of the face. It is also possible to administer botulinum toxic perioperatively. However, care must be taken to minimalize muscle movement in the forehead, and excess tension across a healing wound should be prevented, the scar healing in the area of the wound should be maximized.^[5–7]^ The existing literature shows that studies have confirmed that botulinum toxin type A can reduce wound tension, reduce collagen production and inhibit fibroblasts.^[8]^ A meta-analysis carried out recently showed that that botulinum toxin has the capacity to reduce the width of hypertrophic scars to a considerable extent, which improved the appearance of the patients on visual analogue scales, and improved the overall wellbeing of the patient.^[9]^

Studies that had evaluated the efficiency and safeness of injecting botulinum toxin A for enhancing facial scars is not comprehensive, and the results remain controversial. Therefore, it prompted the undertaking of a meta-analysis to figure out the efficiency and safeness of injecting botulinum toxin A to improve facial scars.

## 2 Methods

The compilation of the protocol conforms with the Preferred Reporting Items for Systematic Reviews and Meta-analyses (PRISMA) guidelines.^[10]^ This study has been registered on open science framework (OSF, https://osf.io/). The registration DOI number of the present study is 10.17605/OSF.IO/94TXP.

### 2.1 Eligibility criteria

#### 2.1.1 Different categories of studies

Randomized or quasi-randomized controlled trials of botulinum toxin A vs no treatment/placebo or another treatment were included.

#### 2.1.2 Included participants

All participants who satisfied the diagnostic criteria of facial scars were included. The participants involved in this study were not filtered by any constraints, such as, age, race, and gender.

#### 2.1.3 Types of interventions and comparisons

1) Experimental interventions
  Botulinum toxin A was administered to patients from the treatment group (no constraints on the dosage and course of treatment).
2) Comparisons interventions
  The comparisons group could gain a placebo, no treatment, another treatment of facial scars, or conventional treatment recommended by a guideline.

#### 2.1.4 Types of outcomes measures

1) Primary outcomes
  The primary outcome measures include Vancouver scar scale (VSS), visual analogue scale (VAS), patient scar assessment scales (PSAS), observer scar assessment scale (OSAS), scar width, and stony brook scar evaluation scales (SBSESs).
2) Secondary outcomes
  The secondary outcome measures include scar color, flexibility, and adverse events.

### 2.2 Search methods for identification of studies

#### 2.2.1 Electronic searches

The electronic databases mentioned below are used to conduct a comprehensive search to find eligible studies. These include PubMed, EMBASE, Web of Science, Spocus, Cochrane Central Register of Controlled Trials, WanFang database, China Nation Knowledge Infrastructure, and VIP database. Regardless of the language and time of publication, all the aforementioned databases shall be searched from inauguration to the present.

#### 2.2.2 Searching other sources

In addition to the databases, Google Scholar and the lists of references will be used to carry out citation tracking of the selected studies for identifying any other eligible studies that could have been missed.

### 2.3 Data collection and analysis

#### 2.3.1 Identification and selection studies

EndNote X9 is used to manage the citations identified through the search strategy. Two independent reviewers will screen the articles for eligibility; the process is done in 2 exclusive steps:

1. citation titles and abstracts and
2. full text.

**Figure 1:**
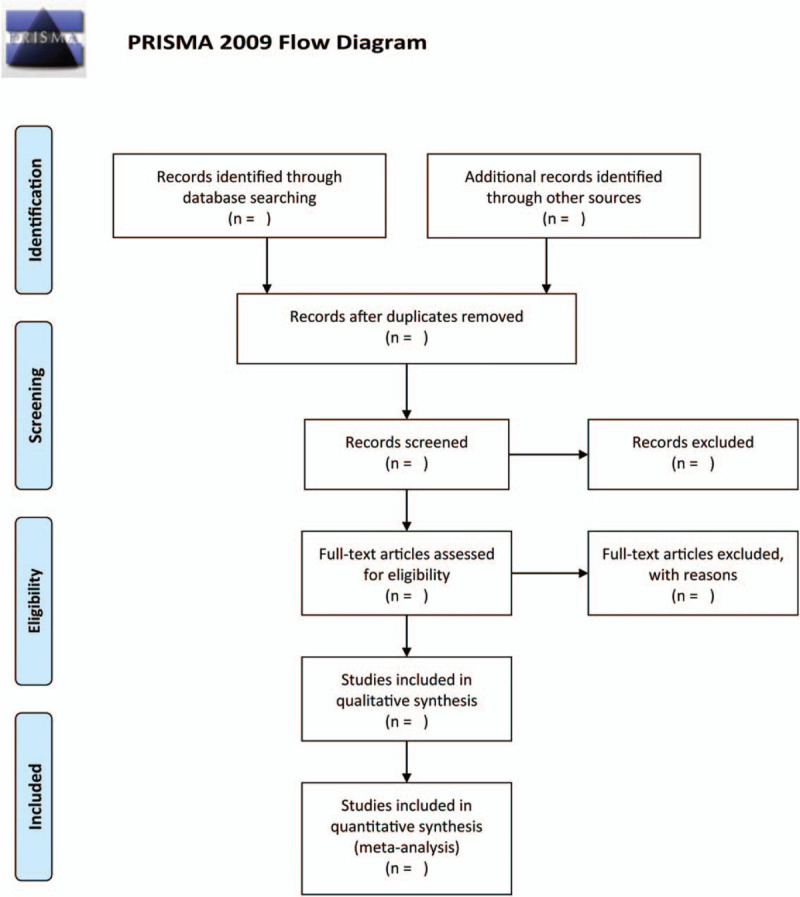
Flow chart of the search process.

Differences that occur in opinion between the 2 reviewers is resolved by consensus or a third reviewer. A flow diagram outlining the process of study selection is illustrated in Figure 1.

#### 2.3.2 Data extraction

The data in the included studies will be extracted by 2 independent reviewers with the aid of an extraction form which was customized in MS Excel. The information includes publication information, study design, patient characteristics, interventions, and outcomes of each study. The form will be pilot-tested on a few articles. Disputes are resolved through consensus or by consulting a third reviewer.

#### 2.3.3 Assessment of study quality

Two reviewers will independently assess the risk of bias in respective outcomes reported in the studies included with the aid of Cochrane Risk of Bias Tool V.2.0.^[11]^

#### 2.3.4 Dealing with missing data

If possible, any unclear or missing data will be obtained from primary authors. In the event where such data cannot be requested, the available data is analyzed by intention-to-treat analysis.

#### 2.3.5 Data synthesis and analysis

Continuous data will be characterized as the mean difference (MD) or standardized mean difference (SMD) with 95% confidence interval (CI) between the 2 groups. Meanwhile, dichotomous data will be characterized as the relative risk (RR) with 95% CI. Assessment of the heterogeneity across the studies adopts the *Q* test and the *I*^*2*^ test. In the case where the value of the *I*^*2*^ test is less than 50%, the fixed effect model will be selected for data synthesis,^[12]^ on the other hand, if the value of the *I*^*2*^ test is between 50% and 75%, the random effects model is selected for synthesising the data.^[13]^ Moreover, if the *I*^*2*^ test value exceeds 75%, the plausible causes of heterogeneity is considered from clinical and methodological perspectives. A detailed analysis or subgroup analyses will be provided. Data consolidation is conducted in RevMan 5.3 (Cochrane, London, UK).

#### 2.3.6 Assessment of reporting bias

If the meta-analysis includes more than ten studies, the publication bias shall be examined by assessing a funnel plot for indicators of asymmetry. Furthermore, statistical investigation will adopt Egger test.^[14,15]^ If asymmetry is identified, plausible explanations shall be provided.

#### 2.3.7 Assessment of sensitivity analysis

If the included trials have significant heterogeneity, sensitivity analysis will be performed to help explore the source of heterogeneity. One study will be deleted at a time before analyzing the remaining studies to estimate if a single study would significantly impact the results.

### 2.4 Ethics and dissemination

Since existing studies are involved, there is no need for an ethics approval.

## 3 Discussion

Considerable advancements have been made on non-operative techniques that can enhance facial scars. Among these, the efficiency of injecting botulinum toxin A to improve facial scars has received considerable interest. Presently, the number of studies that have demonstrated that injecting botulinum toxin A to improve facial scars has had positive clinical outcomes. However, the current status of evidence of botulinum toxin A for treating facial scars have presented controversial outcomes, without any conclusiveness. Therefore, this study aimed to evaluate the efficiency and safety of injecting botulinum toxin A to improve facial scars. This review will add to the existing literature by showing compelling evidence and improved guidance in clinic settings.

## Author contributions

X.D. and H.M.Y. conceptualised the study. Z.D.S., H.X.F., and X.D. drafted the proposal. X.D., Z.D.S., and H.M.Y. designed the study. All authors assisted with manuscript writing and critical revision of the study design and manuscript. All authors read and approved the final manuscript.

**Conceptualization:** Dan Xu, Mengyao hu.

**Data curation:** Dan Xu, Xue-Feng Hu, Mengyao hu.

**Formal analysis:** Dan Xu, Xue-Feng Hu.

**Funding acquisition:** Dan Xu, Da-Song Zhang, Xue-Feng Hu.

**Investigation:** Dan Xu, Da-Song Zhang.

**Methodology:** Dan Xu, Da-Song Zhang.

**Project administration:** Da-Song Zhang.

**Resources:** Da-Song Zhang.

**Software:** Xue-Feng Hu.

**Supervision:** Xue-Feng Hu, Mengyao hu.

**Writing – original draft:** Mengyao hu.

**Writing – review & editing:** Mengyao hu.

## References

[1] Bock O Schmid-Ott G Malewski P Quality of life of patients with keloid and hypertrophic scarring. Arch Dermatol Res 2006;297:433–8. 16528552 10.1007/s00403-006-0651-7

[2] Van Loey NE Van Son MJ Psychopathology and psychological problems in patients with burn scars: epidemiology and management. Am J Clin Dermatol 2003;4:245–72. 12680803 10.2165/00128071-200304040-00004

[3] Boyce ST Lalley AL Tissue engineering of skin and regenerative medicine for wound care. Burns Trauma 2018;6:4. 30009192 10.1186/s41038-017-0103-y PMC6040609

[4] Lighthall JG Fedok FG Treating Scars of the Chin and Perioral Region. Facial Plast Surg Clin North Am 2017;25:55–71. 27888894 10.1016/j.fsc.2016.08.005

[5] Heffelfinger R Sanan A Bryant LM Management of Forehead Scars. Facial Plast Surg Clin North Am 2017;25:15–24. 27888890 10.1016/j.fsc.2016.08.012

[6] Venus MR Use of botulinum toxin type A to prevent widening of facial scars. Plast Reconstr Surg 2007;119:423–4. author reply 424. 10.1097/01.prs.0000245337.38327.80 17255704

[7] Wilson AM Use of botulinum toxin type A to prevent widening of facial scars. Plast Reconstr Surg 2006;117:1758–66. discussion 1767-1758. 16651948 10.1097/01.prs.0000209944.45949.d1

[8] Kasyanju Carrero LM Ma WW Liu HF Botulinum toxin type A for the treatment and prevention of hypertrophic scars and keloids: Updated review. J Cosmet Dermatol 2019;18:10–5. 30548742 10.1111/jocd.12828

[9] Zhang DZ Liu XY Xiao WL Botulinum Toxin Type A and the prevention of hypertrophic scars on the maxillofacial area and neck: a meta-analysis of randomized controlled trials. PLoS One 2016;11:e0151627. 26985661 10.1371/journal.pone.0151627 PMC4795777

[10] Moher D Shamseer L Clarke M Preferred reporting items for systematic review and meta-analysis protocols (PRISMA-P) 2015 statement. Syst Rev 2015;4:1. 25554246 10.1186/2046-4053-4-1 PMC4320440

[11] Higgins JP Altman DG Gøtzsche PC The Cochrane Collaboration's tool for assessing risk of bias in randomised trials. BMJ 2011;343:d5928. 22008217 10.1136/bmj.d5928 PMC3196245

[12] Mantel N Haenszel W Statistical aspects of the analysis of data from retrospective studies of disease. J National Cancer Institute 1959;22:719–48. 13655060

[13] DerSimonian R Laird N Meta-analysis in clinical trials revisited. Contemporary Clin Trials 2015;45:139–45. 10.1016/j.cct.2015.09.002 PMC4639420 26343745

[14] Begg CB Mazumdar M Operating characteristics of a rank correlation test for publication bias. Biometrics 1994;50:1088–101. 7786990

[15] Egger M Davey Smith G Schneider M Bias in meta-analysis detected by a simple, graphical test. BMJ 1997;315:629–34. 9310563 10.1136/bmj.315.7109.629 PMC2127453

